# Chemokine Receptors in Epithelial Ovarian Cancer

**DOI:** 10.3390/ijms15010361

**Published:** 2013-12-31

**Authors:** Goda G. Muralidhar, Maria V. Barbolina

**Affiliations:** Department of Biopharmaceutical Sciences, University of Illinois at Chicago, 833 S Wood Street, PHARM335, Chicago, IL 60612, USA; E-Mail: gmural2@uic.edu

**Keywords:** ovarian carcinoma, chemokine receptor, metastasis, angiogenesis, invasion, migration, proliferation, immune response

## Abstract

Ovarian carcinoma is the deadliest gynecologic malignancy with very poor rate of survival, and it is characterized by the presence of vast incurable peritoneal metastasis. Studies of the role of chemokine receptors, a family of proteins belonging to the group of G protein-coupled receptors, in ovarian carcinoma strongly placed this family of membrane receptors as major regulators of progression of this malignancy. In this review, we will discuss the roles that chemokine-receptor interactions play to support angiogenesis, cell proliferation, migration, adhesion, invasion, metastasis, and immune evasion in progression of ovarian carcinoma. Data regarding the role that the chemokine receptors play in the disease progression accumulated insofar strongly suggest that this family of proteins could be good therapeutic targets against ovarian carcinoma.

## 1. Introduction

Epithelial ovarian cancer is the deadliest gynecological malignancy and a fifth leading cause of death from cancer in women [1]. The American Cancer Society estimates that it will claim 14,230 lives in 2013 [2]. Metastatic progression of EOC is very unique, as metastasis that cause death spreads loco-regionally throughout the peritoneal cavity [3]. Malignant cells are shed off of the primary tumor and are carried by the intraperitoneal ascitic fluid, which is followed by implantation at the organs and tissues of the peritoneal cavity, anchorage in sub-mesothelial extracellular matrix and establishment of metastases [4–6]. Complex signal transduction mechanisms underlie processes of angiogenesis, migration, and invasion, involved in metastatic progression of ovarian cancer. These processes are regulated in part by the seven trans-membrane domain receptors known as G protein-coupled receptors (GPCRs), an abundant class of proteins covering an estimated 4% of the entire protein-coding genome [7–10].

Human GPCRs are grouped into six classes based on sequence homology and functional similarity, including classes A (rhodopsin-like), B, C, D, E, and F [11–14]. The rhodopsin family is further sub-classified into α, β, γ and δ families [15]. Chemokine receptors belong to the γ rhodopsin family of GPCRs and are activated by both secreted and membrane-tethered protein-ligand chemokines, also known as chemo-attractant cytokines [15,16]. Structurally, all chemokines share a common monomeric structure consisting of a flexible *N*-terminus followed by an *N*-loop, three anti-parallel β-strands and a *C*-terminal α-helix [17–19]. The *N*-terminal domain contains one or two cysteines implicated in structure-stabilizing intra-molecular disulfide bonds. The chemokines are divided into four groups: C, CC, CXC and CX_3_C based on the position of these cysteines [20]. Accordingly, chemokine receptors are named CR, CCR, CXCR or CX_3_CR. The C chemokines lack cysteines one and three of the typical chemokine structure. Members of the CXC group are characterized by the presence of a single amino acid between the first two *N*-terminal cysteines, while members of the CC class lack this amino acid. In CX_3_C chemokines, three amino acids are present between the first two cysteines [20,21].

Chemokines interact with their cognate receptors according to a two-step model [17,22,23]. The initial step involves anchoring of the chemokine ligand to the *N*-terminus of the receptor and is followed by the binding of the flexible chemokine *N*-terminus to the extracellular loops and the *trans*-membrane segments of the receptor. Binding of a chemokine ligand to its receptor leads to a conformational changes in the receptor that allow binding and activation of a trimeric G protein, leading to the initiation of downstream signal transduction pathways that regulate migration, adhesion, proliferation, and metastasis in cancer cells [24–28]. Some chemokines can bind and activate several different receptors, and many chemokine receptors can be activated by multiple various chemokine ligands, while other chemokines, such as CXCL12, XCL1/2, and CX_3_CL1 bind and activate only their specific receptors CXCR4, XCR1, and CX_3_CR1, respectively. Interestingly, studies suggest that binding of different chemokines to the same receptor can produce different functional outcomes [29], thus emphasizing a potential complexity of the chemokine-receptor signaling. Hence, detailed understanding of the signal transduction mechanisms triggered by chemokine-receptor interactions could be essential to developing drugs targeting these interactions in order to prevent their functional outcomes in a disease. To add to the complexity of chemokine-receptor signaling, chemokine receptors may undergo constitutive homo- or hetero-dimerization, which may promote cross talk between different signal transduction pathways [24,30–33]. For example, a cross talk between CXCL12-activated CXCR4 and epidermal growth factor receptor (EGFR) has been proposed to link cell proliferation signals in ovarian carcinoma [34,35].

**Figure 1.**
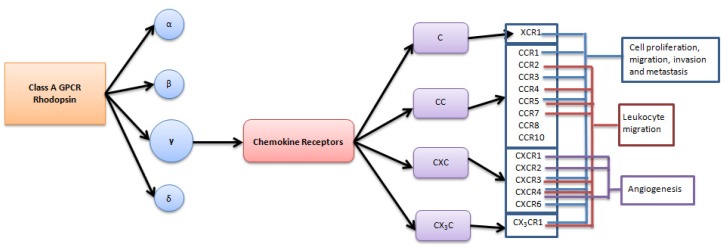
Chemokine receptors shown to play a role in progression of ovarian carcinoma.

Cell proliferation, adhesion, migration, angiogenesis, and immune evasion are some of the hallmarks of cancer, which is necessary to ensure success of growing tumors and disseminating metastasis [36]. Recently, multiple studies have linked chemokine-receptor axes with angiogenesis, immunosuppression, invasion, migration, and proliferation in ovarian carcinoma, as well as established their role in homing metastasizing cells to their niches in the peritoneal cavity (Figure 1).

## 2. Role of Chemokine Receptors in Regulation of Angiogenesis in Ovarian Carcinoma

**Figure 2.**
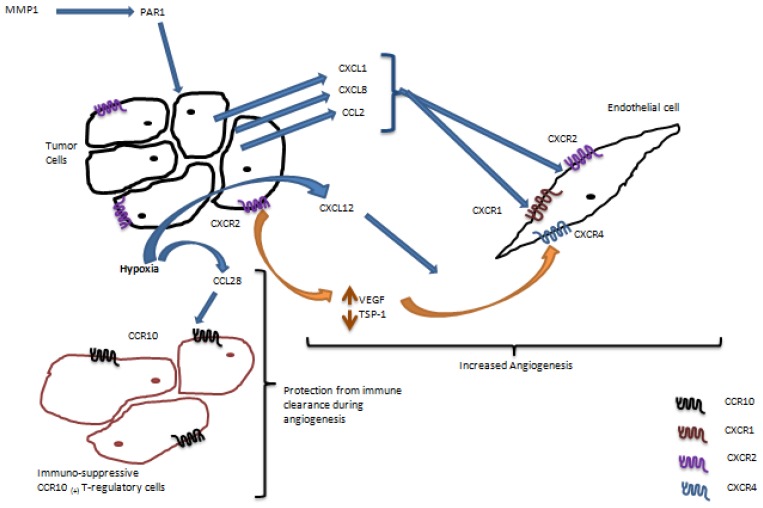
Chemokine receptors shown to support angiogenesis in ovarian carcinoma.

Angiogenesis is one of the prominent characteristics of many solid cancers, including ovarian carcinoma, as growing peritoneal metastasis require constant supply of oxygen and nutrients to support their rapid growth [37–39]. Paracrine interactions between chemokines produced by the ovarian cancer cells and chemokine receptors expressed by the endothelial cells have been shown to stimulate angiogenesis (Figure 2). CXCR1 and CXCR2 are the two main chemokine receptors expressed by the endothelial cells that mediate angiogenic response [40]. Protease activated receptor-1 (PAR1) is a G-protein coupled receptor that is activated by proteolytic cleavage of its extracellular domain [41]. It has been shown that matrix metalloproteinase-1 (MMP-1) causes activation of PAR1 leading to expression of the pro-angiogenic secreted chemokines CXCL8 (IL-8), CXCL1 (GRO-α), and CCL2 (MCP-1) by the cancer cells [42]. These chemokines bind to the CXCR1 and CXCR2 receptors expressed by the endothelial cells and induce endothelial cell proliferation, migration, and tube formation. Increased CXCL8 has been detected in the serum of patients with ovarian cancer [43], and more recently it has been associated with poor clinical outcome and worse survival in patients with this malignancy [44]. Expression of CXCR2 by the ovarian cancer cells themselves has also been demonstrated, and it has been shown that it can promote angiogenesis by increasing levels of vascular endothelial growth factor (VEGF) and decreasing levels of thrombospondin-1 (TSP-1) via a mitogen-activated protein kinase (MAPK) and a signal transducer and activator of transcription 3 (STAT3) dependent mechanisms [45].

VEGF, which is essential for angiogenesis, has been shown to up-regulate expression of CXCR4 in vascular endothelial cells [46]. Furthermore, hypoxia can induce expression of the CXCR4 ligand CXCL12 by the tumor cells [46]. Thus, angiogenesis in ovarian cancer may be potentiated by the interaction of CXCL12 produced by the cancer cells and CXCR4 expressed by the endothelial cells in hypoxic conditions. Importantly, it has been shown that disruption of the CXCR4/CXCL12 axis using CXCR4-specific RNAi or a small molecule inhibitor of CXCR4 AMD3100 led to a decreased tumor vessel density and markedly reduced tumor angiogenesis in a FVB/NJ immunocompetent model of ovarian cancer [47].

Hypoxic stress in the tumor microenvironment during angiogenesis can cause release of the damage-associated molecular pattern molecules (DAMPs) [48]. These molecules can trigger immune clearance of the tumors [48]. In order to overcome cancer cell clearance by the immune system during angiogenesis, ovarian cancer cells have developed an ingenious mechanism [49]. Tumor hypoxia can robustly induce expression of the CC-chemokine ligand 28 (CCL28) by hypoxia inducible factor 1α (HIF1α) dependent mechanism [49]. The receptor for CCL28, CCR10, is expressed by a population of regulatory T cells, a subpopulation of T-cells that suppresses immune response of T-cells [49,50]. It has been shown that increased expression of CCL28 during angiogenesis allows recruitment of the CCR10^+^ T-regulatory cells, which protect tumor cells from immune clearance and increase tumor angiogenesis and vascularization [49].

## 3. Role of Chemokine Receptors in Regulation of Ovarian Carcinoma Metastasis

**Figure 3.**
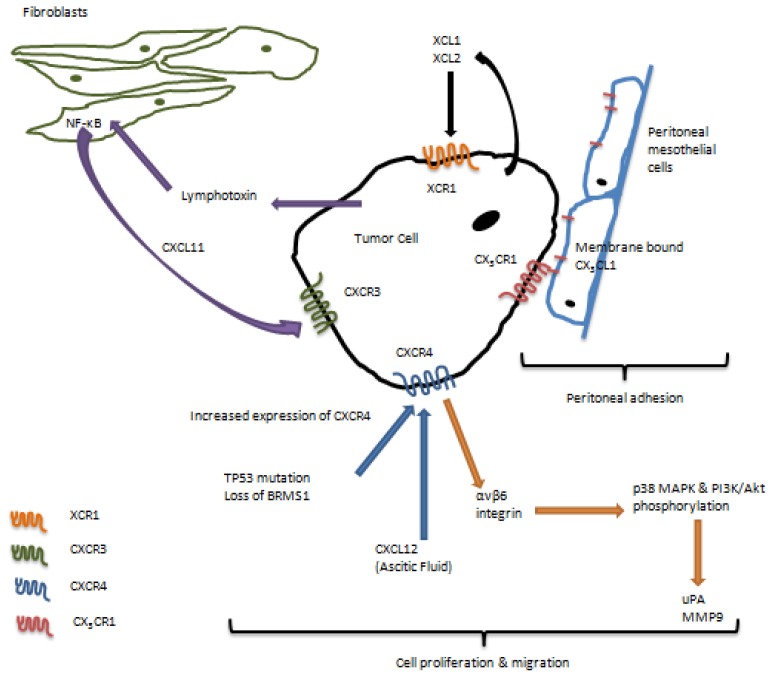
Chemokine receptors shown to play a role in cell proliferation, adhesion, migration, invasion, and metastasis in ovarian carcinoma.

The metastatic phenotype requires cell proliferation, adhesion, migration, and invasion, among other properties, to ensure successful colonization of the secondary organs. Metastasizing ovarian carcinoma cells shed from the primary tumor attach to mesothelial cells covering peritoneal tissues and organs following adhesion to and retraction of the mesothelium [4,51–53]. Cell proliferation has a prominent position in development and progression of ovarian carcinoma, as metastatic lesions are known to reach very large sizes, especially in comparison with the primary tumor. Cell migration and invasion support growing primary tumors and metastasis by ensuring anchorage of metastatic lesions in peritoneal tissues and organs. Many studies have shown a role for chemokine receptors in supporting cell proliferation, adhesion, migration, and invasion, which ultimately could lead to regulation of peritoneal metastasis (Figure 3).

We have recently shown that a chemokine receptor fractalkine CX_3_CR1, a single member of the CX_3_C group of chemokine receptors, is expressed in primary and metastatic ovarian carcinoma [54]. CX_3_CR1 is activated by a single ligand, fractalkine (CX_3_CL1), which is a unique chemokine, as it may be present in soluble and membrane-tethered forms [55]. We have found that the peritoneal mesothelial cells express the membrane-bound form of CX3CL1, and ovarian carcinoma cell adhesion to the mesothelial monolayer was significantly impaired in the presence of the CX_3_CL1-specific function blocking antibodies as well as upon downregulation of CX_3_CR1 in ovarian cancer cells using siRNA [54]. These data indicated that the fractalkine axis in ovarian carcinoma could play a major role in regulation of peritoneal adhesion, further suggesting that abrogation of the CX_3_CL1/CX_3_CR1 interaction in ovarian carcinoma may reduce metastatic burden by blocking formation of the secondary lesions by preventing attachment and adhesion of the metastasizing cells to the organs and tissues of the peritoneal cavity. Furthermore, our studies indicated that the CX_3_CL1/CX_3_CR1 axis supported ovarian carcinoma cell adhesion and proliferation, further suggesting its potential pivotal role in development and progression of ovarian carcinoma metastasis. Importantly, it has been found that ovarian carcinoma cells themselves express the fractalkine ligand [56]. Thus, expression of both fractalkine receptor and its ligand may indicate a potential importance of this axis in progression of ovarian carcinoma.

Our group has recently shown that the lymphotactin receptor XCR1, the only member of the C family of chemokine receptors plays an important role in metastatic development of ovarian cancer [57]. This receptor, which is not expressed in the normal ovarian tissue, gains expression in primary and metastatic human epithelial ovarian carcinoma. Chemokines lymphotactin 1 and lymphotactin 2, XCL1 and XCL2, respectively, bind and activate XCR1, leading to increased proliferation and migration of ovarian cancer cells that express XCR1. The ovarian carcinoma cells themselves express XCL1, and XCL2 is detected in the ascites from ovarian carcinoma, suggesting that XCL/XCR1-driven cell proliferation and migration could contribute to growth and expansion of peritoneal metastasis. Importantly, using a xenograft mouse model with intraperitoneally injected ovarian carcinoma cells we showed that ovarian carcinoma dissemination to the peritoneal wall and diaphragm strongly depended on XCR1 expressed by the ovarian cancer cells, suggesting the presence of lymphotactin-dependent mechanisms supporting metastatic cell homing to these sites [57].

Stromal cells in the tumor microenvironment have recently emerged as major regulators of the malignant phenotype [58–60]. Interestingly, the underlying mechanisms were demonstrated to be mediated in part by the chemokine-receptor networks. Primary tumor cells can produce a cytokine lymphotoxin, which stimulates the release of a chemokine CXCL11 from the neighboring stromal fibroblasts though a lymphotoxin beta receptor - NFκB signaling dependent mechanism [61]. CXCL11 binds to its cognate receptor CXCR3 overexpressed by the tumor cells, which could promote cell proliferation and migration [61]. Additionally, overexpression of CXCR3 is significantly associated with the tumor grade and lymph node metastasis, suggesting a role for CXCL11/CXCR3 in promoting ovarian carcinoma metastasis [61].

C-X-C chemokine receptor-4 (CXCR4) belongs to a CXC family of chemokine receptors and is activated by a single ligand CXCL12 specific to this receptor. High grade serous ovarian cancer (HGSOC), the deadliest type of the disease [62], contains focal genomic amplification of the CXCR4 gene locus along with the mutations in TP53, suggesting that those may be some of the early events in the development of HGSOC, and they could contribute to chromosomal instability of ovarian carcinoma cells [63]. Others and we have found that CXCR4 is overexpressed in specimens of human ovarian cancer, and its specific ligand CXCL12 is present in the ascitic fluid collected from patients with ovarian carcinoma [63–69]. The NFκB signaling may regulate the expression of CXCR4 in ovarian carcinoma by multiple mechanisms, including those involving three-dimensional collagen type I (as a model of the ECM present at the metastatic sites) and via loss of the breast cancer metastasis suppressor 1 (BRMS1) [67,69]. It has been shown that CXCL12 elicits intracellular calcium influx, resulting in chemotaxis, increased cell proliferation, and changes in β1 integrin expression in ovarian cancer cells [66,70]. It has been recently found that CXCL12 enhances cell invasion by a αvβ6 integrin signaling dependent mechanism through downstream effectors p38 MAPK and PI3K/Atk resulting in upregulation of urokinase-type plasminogen (uPA) [68]. Furthermore, the cross-talk between CXCR4 and EGFR may augment expression of matrix metalloproteinase 9 (MMP-9) [65]. In support of the role of CXCR4 in regulation of expression of proteolytic enzymes, knockdown of CXCR4 decreased the secretion of proteolytic uPA and MMP-9 [71]. Of note, elevated MMP-9 expression was caused by an interaction of CCL5 and its receptors CCR1, CCR3 and CCR5 via the NFκB signaling pathway in ovarian cancer stem-like cells [72]. Expression of CXCR4 correlated with reduced survival of patients with ovarian carcinoma [64]. Importantly, studies involving a FVB/NJ immunocompetent mouse model of ovarian carcinoma as well as the use of CXCR4-specific RNAi and AMD3100 have shown that abrogation of CXCR4 robustly decreased intraperitoneal metastasis and almost completely abolished metastasis to the omentum, one of the major sites colonized by ovarian carcinoma [47]. Additionally, abrogation of CXCL12/CXCR4 using AMD3100 reduced peritoneal dissemination in a xenograft mouse model as well [73]. Increased tumor cell apoptosis and necrosis has also been observed following impairment of the CXCL12/CXCR4 axis [47]. In another study a peptide antagonist of CXCR4, CTCE-9908, was able to induce cell death by mitotic catastrophe via multinucleation, G2-M arrest, and abnormal mitosis [74]. To add more complexity, it has been found that CXCL12 can bind and activate another chemokine receptor, CXCR7 [75], however, neither expression nor function of this receptor has been described in ovarian carcinoma.

Along with CXCR4, the CXCR6 receptor and its ligand CXCL16 were reported to be significantly upregulated in ovarian cancers compared to the normal ovarian epithelium or benign ovarian tumors [64]. Expression of both CXCR4 and CXCR6 strongly correlated with lymph node metastasis, and expression of CXCL16 correlated with reduced patients’ survival [64].

## 4. Chemokine Receptors and the Immune System

T-cells often infiltrate ovarian carcinoma, and the presence of intratumoral T-cells has been associated with better outcome [76,77]. However, ovarian carcinomas can develop mechanisms of tolerance or immunosuppression that allow them evading the harmful action of the immune system [78–82]. Chemokine-receptor networks facilitate some of these underlying mechanisms. It has been shown that the ovarian cancer cells, as well as associated macrophages and myeloid dendritic cells produce chemokine CCL22, a ligand for CCR4 [83,84]. CCR4 is expressed by a subpopulation of immunosuppressive T regulatory cells. CCL22, secreted by the tumor cells causes accumulation of CCR4^+^ T-regulatory cells at tumor site resulting in immune suppression [84]. It was also shown that the circulating T-regulatory cells display significantly higher expression of CCR4 than the tumor infiltrating T-regulatory cells suggesting that they could be recruited via the CCR4/CCL22 axis [83].

It has been shown that a subpopulation of the FOXP3^+^ T-regulatory cells, which express CXCR3, is more prevalent among the tumor associated as well as tumor infiltrating lymphocytes [85]. These T-regulatory cells accumulate in the ovarian cancer microenvironment due to the release CXCL10, a ligand for CXCR3, by the cancer cells. The CXCR3^+^ T-regulatory cells suppress the proliferation of T-effector cells and the production of interferon γ (IFN γ), thereby limiting type I immunity. Further characterization of the regulatory T cells showed the presence of an immune suppressive CD8^+^ subpopulation, which was CCR7^+^ and migrated in response to the gradient of CCL19 [86]. These cells are induced by the macrophage derived plasmacytoid dendritic cells present in the tumor ascites [86].

It has been shown that the CCL2/CCR2 axis is a determinant of the degree of macrophage infiltration in ovarian cancer [87–90]. The CCR2 receptors expressed by the monocytes facilitate monocyte migration into the tumors in response to gradients of CCL2 [89]. Following their entry into the tumor the monocytes develop a selective defect in CCR2 expression, which prevents the chemokine scavenging function of the monocytes while keeping them arrested within the tumor [89]. Macrophages isolated from both the ascitic fluid and solid tumor from ovarian cancer patients exhibited very low levels of CCR2 mRNA, which correlated with the lack of chemotactic response to CCL2 [89]. The defective CCR2 expression was regulated by the local tumor necrosis factor alpha (TNF-α) [89]. TNF-α has been associated with increased tumor grade in ovarian cancer [91]. In addition to its role in CCR2 expression, TNF-α also stimulated up-regulation of CXCR4 [92]. This pathway was also involved in the increase CXCR4 mRNA expressed by the cancer cells upon their co-culture with macrophages [92]. The CXCL12/CXCR4 axis has also been shown to play a role in recruiting T-regulatory and plasmacytoid dendritic cells [47,93,94]. Prostaglandin E2 can induce CXCR4 expression in the myeloid derived suppressor cells (MSDCs), which may be subsequently recruited into the tumor by CXCL12 present in the ascitic fluid [93]. It has also been shown that CXCR4 inhibition significantly reduces T-regulatory cell infiltration into the tumor while increasing antitumor immunity [47].

Development and dominance of different subsets of monocytes within the peritoneal leukocyte population during cancer progression has been shown using transplantable models of murine ovarian cancer [95]. Early in the process, CX_3_CR1^low^ and GR-1^high^ cells dominated within the CD11b^+^ cell population, and later increase of the CX3CR1^high^ population has been detected. For both of these populations CCR2 served as a facilitator for their recruitment into the peritoneum wherein they could exert their immunosuppressive effect on the naïve CD8^+^ and CD4^+^ T cells [95].

The chemokine receptor signaling in the helper NK cells is involved in recruiting T effector cells to the tumor microenvironment [96]. It was shown that the helper NK cells stimulated by IL-18 produced CXCL9, CXCL10 and CCL5 that attracted immature dendritic cells expressing their receptors CXCR3 and CCR5 [96]. This was followed by the recruitment of effector T cells, which promote type I immune response against cancer [96,97].

Adoptive transfer of anti-tumor T-cells has been proposed as a mechanism to suppress endogenous anti-tumor immunity for better treatments of this deadly disease. Use of a short *ex vivo* priming process in which the naïve T cells were treated with tumor antigens for about 7 days has been reported [97]. This resulted in overexpression of CCL5, the ligand for the CCR5 receptor, by the T-cells. CCR5 is highly expressed by the dendritic cells that have potent immune stimulatory effects. Following injection of these T cells the CCR5^+^ dendritic cells were recruited into the tumor microenvironment by CCL5 and elicited a prolonged endogenous anti-tumor response [97].

Better understanding of the molecular players involved in the leukocyte function within the tumors may facilitate re-establishing the anti-tumor immunity.

## 5. Chemokine Receptor Inhibitors

As highlighted in this review, studies of ovarian carcinoma indicated that chemokine receptors cumulatively play a major role in progression of the disease by contributing to angiogenesis, cell proliferation, adhesion, migration, invasion, and immune evasion. Other cancers along with non-cancerous pathologies also depend on various chemokine-receptor axes [98–102]. Chemokine receptors are membrane-bound proteins, which could be targeted therapeutically. Hence, chemokine receptors have long been considered good therapeutic targets against many pathological conditions [103–105]. Following extensive preclinical studies for chemokine receptor antagonists, about 40 drug candidates have progressed into clinical trials for diseases including AIDS, rheumatoid arthritis, multiple sclerosis, atherosclerosis, asthma and others [106]. These efforts resulted in the approval of two drugs [106], such as a CCR5 inhibitor Maraviroc (Pfizer) [107] and a CXCR4 antagonist, Plerixafor or AMD3100 (Anormed) [108,109]. Importantly, clinical trials evaluating efficacy of small molecule inhibitors as well as antibodies against different chemokine receptors for T-cell leukemias [110], lymphomas [111], multiple myeloma [112], pancreatic [113], and laryngeal cancers [114] are currently underway. The outcomes of these trials will affect future studies of the role of chemokine receptors in ovarian carcinoma and their application to treatment of the disease.

## 6. Conclusions

This review highlights the importance of chemokine-receptor interactions in ovarian cancer as supporters of many functions necessary for tumor progression. The complexity of these interactions is heightened as chemokine receptors are expressed by several cell types in the ovarian cancer microenvironment. These include the cancer cell themselves, as well as stromal cells, endothelial cells and multiple types of leukocytes. Further studies of the diverse roles played by the chemokine-receptor axes in ovarian carcinoma are crucial for rational development of novel therapeutic modalities against epithelial ovarian cancer.
